# HIV Vulnerability Among Survival Sex Workers Through Sexual Violence and Drug Taking in a Qualitative Study From Victoria, Canada, With Additional Implications for Pre-exposure Prophylaxis for Sex Workers

**DOI:** 10.3389/fsoc.2021.714208

**Published:** 2022-01-03

**Authors:** Bryan Eric Benner

**Affiliations:** Department of Sociology, University of Victoria, Victoria, BC, Canada

**Keywords:** hiv/aids, intimate partner violence, sexual violence, sex work, grounded theory, criminology, substance use, PrEP

## Abstract

**Objective:** This qualitative study investigates how social and structural forces mediate vulnerability to HIV infection and transmission among survival sex workers, their clients, and their non-commercial, intimate partners—with especial focus on sexual violence and drug taking.

**Method:** I employed an adapted grounded theory approach to conducting and analyzing (*n* = 9) open-ended, in-depth interviews with a convenience sample of currently working (and recently exited) survival sex workers from a community setting in Victoria, Canada.

**Findings:** Participants revealed important contexts and conditions under which they were vulnerable to HIV infection. At the behavioural level, participants were aware of how HIV could be transmitted (condomless sex and sharing drug equipment), yet participants voiced strongly how structural and systemic features (for instance, client violence, the need for drugs, and “bad date” referrals) could squeeze and constrain their agency to take up safer practices, mediating their optimal HIV health and safety. Some participants reported strained relationships with police because of previous drug involvement.

**Conclusion:** Survival sex workers constitute a health population vulnerable to HIV infection, and ensuring there could be a supportive (outreach) community replete with HIV resources is paramount. The availability of safer sex and drug equipment play important roles in HIV behavioural prevention efforts. However, uptake of pre-exposure prophylaxis (PrEP) at no cost in the Canadian province of British Columbia could be an important and beneficial structural intervention for non-injection drug taking cis-female sex workers in this study who are presently ineligible for no cost PrEP.

## Introduction

### Background and Study

Survival sex workers have been recognised across the literature as an intensely stigmatised health population whose members live and work amid myriad contexts and conditions that drive sexual violence and raise their risk of HIV infection (Shannon et al., 2008; Chettiar et al., 2010; Landsberg et al., 2017; Argento et al., 2020). The precise social demographics of the sex work community in Victoria, Canada remain speculative, but within any year, roughly 1,000 adults trade and sell sex on a fulltime or part-time basis (Benoit et al., 2017a). The term, “survival sex” “emphasises the precarious economic position and disempowerment of those who engage in it, and the absence of other means of generating income” (McMillanet al., 2018:1,523). Very little is known about how survival sex workers living *without* HIV in the Victoria community conceptualise and characterise their vulnerability to HIV infection and transmission.

The following three research questions guided this study: Firstly, how are social contexts and conditions understood to mediate HIV vulnerabilities among survival sex workers living *without* HIV when offering commercial services to clients? Secondly, what are the social contexts and conditions under which survival sex workers perceive greater HIV vulnerabilities with their non-commercial, intimate partners? And lastly, what are the strategies employed by sex workers to maintain and support their sexual health, and how are these strategies understood?

In answering these questions, I examine a rich qualitative dataset generated from participant interviews (*n* = 9) with cis-male and cis-female (currently working and recently exited) survival sex workers living *without* HIV from a community setting in Victoria, Canada. I proceed with a social health exploration of how these sex workers understand and experience HIV vulnerabilities, and I give additional attention towards the their strategies to monitor and maintain their HIV health and safety. I use these dimensions to invigorate a trauma-informed discussion with especial considerations for HIV health literacies. The knowledge from this study could provide a backdrop for discussions on social policy and programming [for instance, the no cost eligibility for pre-exposure prophylaxis (PrEP)] aimed at ameliorating the health, safety, and well-being of this vulnerable health population.

### Architecture of Article

I begin in the following section by examining legislation concerning the sex trade in Canada. I then highlight the often-adversarial role of the police within the sex trade, and I follow this by reviewing alcohol and drug taking in the sex trade. I review occupational risk environments in the sex trade. I then present the adapted grounded theory methods and results. At the interpersonal and behavioural levels, participants were aware of how HIV could be transmitted (condomless sex and sharing drug equipment). Participants voiced how structural and systemic features (for instance, client violence, the need for drugs, and “bad date” referrals) constrained their agency to meet the paragon for safer practices. I consider in the discussion section the availability and deployment of no cost pre-exposure prophylaxis (PrEP) for eligible program participants within the Canadian province of British Columbia (BC). I conclude with a brief summary, and I discuss limits to this study.

## Sex Work and HIV Vulnerabilities

### Sex Work Law and HIV Vulnerability

Health scholars have found that the criminalisation of sex work in Canada reduces control over occupational conditions, scales up violence and discrimination, marginalises sex workers from health care and social services, and drives sex work to unsafe spaces or underground (Shannon et al., 2008; Shannon et al., 2009a; Shannon and Csete, 2010; O’Doherty, 2011; Platt et al., 2018; Mangat et al., 2021).

The selling of sex is recognised as legal employment in Canada; however, core occupational features that could otherwise enhance the health and safety of sex workers remain against the law. Canada’s *Bill C-36* from November of 2014 criminalises the following: obtaining (or communicating for) sexual services, receiving third-party financial or material benefit from sexual services, and communicating for sexual services anywhere persons under 18 could be present (*S.C.* 2014, c.25). In short, the selling (and trading) of sex remains legal for sex workers, but the “purchase” of sexual services by their clients is illegal. This approach mirrors the Nordic model whereby the “end demand” side of the sex trade (largely heterosexual males) is criminally targeted, while the “supply” side (largely heterosexual females) becomes “encouraged” to exit, in theory at least (Johnson and Matthews, 2016). Scholars have been unable to identify improvements to the health and safety of sex workers in the BC in the wake of drafting *Bill C-36*. For instance, in Vancouver, the sexual violence, distrust of law enforcement, and displacement to unsafe neighbourhoods that constrain sex workers’ agency in safer sex negotiation and client screening have been the same both before (Shannon et al., 2008) and after (Landsberg et al., 2017; Krüsi et al., 2014; Krüsi et al., 2016) the “end demand” of the sex trade became criminally targeted.

### Police Patrolling and the Spatial Displacement of Sex Workers

The present opioid epidemic, a “mass poisoning” event on account of an unsafe drug supply (Tyndall, 2020), has been especially harmful for vulnerable populations in BC (Salters et al., 2021), and antagonistic policing practices have been demonstrated to undermine lifesaving overdose prevention services—and exacerbate the risks of overdoses for vulnerable women (Goldenberg et al., 2020). Unsafe occupational environments are powerful factors driving HIV infection risks among sex workers (Benoit and Millar 2001; Shannon et al., 2008; Krüsi et al., 2016). According to Krüsi et al. (2016), the violence, theft, and fraud that is committed *against* survival sex workers remains often responsibilised (to the sex worker) and normalised by police as part and parcel with working in the sex trade; sex workers remain consequently reluctant to report crimes (Krüsi et al., 2016; see also; Crago et al., 2021).

Widespread repressive policing practices are inextricably interwoven with HIV and ST/BBI infection and transmission (Platt et al., 2018). Intense stigma and the framing of sex workers by police as both “victims” and “victimisers” drive adverse interactions between survival sex workers and law enforcement officials in BC (Krüsi et al., 2016) and other global regions (Rhodes et al., 2008; Shannon and Csete, 2010). The principal causes of distrust of police are perceived stigma and discrimination (Benoit et al., 2016).

Gentrification has played an important role in moving “outdoor” sex transactions “indoors” wherein the use of online technologies for sex transactions proliferates (Argento et al., 2020; see also; Lyons et al., 2017). For instance, in the Yaletown area of Vancouver, the former “Boystown” was largely extinguished by urban planning prior to the *2010 Winter Olympics* and, consequently, online sex transactions among male sex workers increased (Argento et al., 2020). On one hand, these online sex transactions decreased stigma and reduced harassment from police, and the online platforms accommodated greater screening of clients through webcams prior to meeting for sex (Argento et al., 2020). On the other hand, the shift to online platforms ruptured longstanding social networks among street-based sex workers, increasing their perceived isolation, scaling up competition, and bringing new vulnerability in the forms of “fake” online profiles created by clients (Argento et al., 2020). Neighbourhood renewal can increase the presence of private security and police, constraining the agency of sex workers to remain in “safer” public spaces (Shannon et al., 2016; Hubbard 1998)—part of a greater structural project of spatial containment, exclusion, and surveillance of sex workers (Hubbard, 2016; Laing and Cook, 2014).

### Alcohol and Drug Taking in the Sex Trade

Drug taking is common among survival sex workers (Chettiar et al., 2010; Landsberg et al., 2017; Deering et al., 2011). In Victoria, Benoit and Millar (2001) found that roughly one in five sex workers’ initial entry in the sex trade was driven by the need for drugs and alcohol; in the previous 6 months, current sex workers reported taking crack and cocaine (48.2%), opioids (36.6%), and meth (9.0%). Crack and cocaine were more common among females, while meth was more common among males; drug taking was altogether more common among street-based (outdoor) sex workers than indoor and independent sex workers (Benoit and Millar, 2001).

Several functions of drug taking have been reported by sex workers in Victoria (Benoit and Millar, 2001) and globally (De Graaf et al., 1995). For instance, drug taking while working longer shifts can bolster energy and offer an escape from the more problematic aspects of sex trade realities (Benoit and Millar 2001) while also allowing some sex workers to overcome aversion to their clients (De Graaf et al., 1995). In the present opioid epidemic in BC, drug taking can be understood as largely “demand-driven” through self-medicating, especially for persons who have experienced trauma, emotional pain, isolation, and mental health challenges, among other factors (Tyndall, 2020). I proceed in the following section with the methods guiding the present study.

## Methods

### Participants and Procedures

The setting for this study, the Greater Victoria area, has a population of 367,770 persons (Statistics Canada 2016). The author conducted interviews in July and August of 2015. Cis-male (*n* = 2) and cis-female (*n* = 7) interview participants constitute the sample for this study. The community non-profit organisataion, *PEERS*, assisted in participant recruitment through a convenience sampling procedure (word-of-mouth). *PEERS* promotes sex workers’ community health and safety through advocacy, research, and evidence-based approaches.

Participants in this study were selected because they had serviced clients on at least 15 occasions in 1 year, they were at least 19 years of age, and they were legally entitled to work in Canada. Participants were HIV seronegative or uncertain of their HIV status at the time of the interviews. The *Human Research Ethics Board* at the University of Victoria approved this study (Appendix 1 for thematic interview guide). After reviewing the purposes and scopes of this study with participants, consent for participation was obtained verbally. Interviews were audio-recorded on a password encrypted recording device, and verbatim interview transcripts were secured on a password-encrypted MS Word file on password-encrypted Windows computer. The interviews lasted for roughly 1 hour each. Participants were debriefed with resources concerning HIV and sex work vulnerability at the close of the interviews.

### The Grounded Theory Approach and Data Analysis

This study adapted a constructivist grounded theory approach (see Charmaz 2006) in the recruitment of a convenience sample (rather than a “theoretical” sample) due to the difficult-to-reach nature of this health population. This grounded approach followed a subjectivist epistemology and relativist ontology (Charmaz, 2006). The study began with a rapid review of the literature to inform the thematic interview guide; interviews were transcribed verbatim and uploaded to the software program, *Dedoose*, for qualitative analysis. Firstly came initial codes, simple line-by-line coding—accompanied by intense memo-writing. Then came categorical and concept codes. These codes were mapped and interrelated, and greater themes were developed. Theoretical saturation occurred at approximately the seventh interview; here, the inclusion of additional participant interview data resulted in no further development and refinement of core categories and themes. The author conferred with colleagues listed in the “acknowledgements”, that the interview data were adequate at *n* = 9 participants. Underscoring analysis was an impetus to preserve participants’ voices through the use of big block quotes. The final stage of this research process was the complete literature review (Charmaz 2006).

## Results

### HIV Vulnerability—An Overview

Interview participants more often characterised their understandings of behavioural HIV vulnerabilities in relation to (protective) condom use and (safer) drug taking. HIV health promotion discourse resonated strongly in participants’ narratives: Participants were aware that HIV transmission could occur only through the exchange of bodily fluids. Participants’ narratives also captured structural and environmental drivers of HIV vulnerability surrounding their drug taking and condom use. HIV vulnerabilities within client and non-commercial, intimate partnerships contrasted: On one hand, the analysis revealed strong social and economic forces driving riskier practices in sex trade engagement with *clients,* whereas participants overall expressed greater agency in the aversion or dismissal of perceived HIV and ST/BBI vulnerability with their *non-commercial, intimate partners*.

All participants in this study had previously worked “independently” at some point. Some participants had worked previously under a “pimp” or manager. There was no indication participants had engaged in occupational environments that offered greater safety and security over time. Participants voiced how riskier sex practices with clients occurred more often when there was an extreme need for drugs (including dope-sickness) and money (especially to support childcare). Participants recalling their street-based sex work had emphasised their history of “rape” and more broadly sexual violence often in the contexts of drugs. Lastly, two interview participants shared that they had never been on “bad dates” whereby their occupational health and safety—and general agency to take up safer practices—would be compromised.

### Responsibilising “Other” Sex Workers for Client HIV Risks

H.B offered a unique perspective on HIV transmission vectors. Intense social and economic forces drove H.B.’s (*female, 30*) engagement in survival sex work, especially the need for drugs. The perceived risk of HIV from her clients ultimately came from “other” sex workers’ riskier HIV practices. “Other” female injection drug taking sex workers were at the foreground in H.B.’s HIV risk narrative:

I know that other hookers mostly have diseases by using IV heroin. I have to use protection (condoms) when I go on dates so that I don’t get HIV … it’s the sickest thing. Other girls do rigs. But hookers like me, I don’t do rigs. Other hookers positive their johns, and they can positive me by sleeping with me without a condom. So, now when a guy picks up a girl and she has diseases, now he could give them to me because he’s mad at her. And it’s not fair, because I didn’t do anything. But the other girl did, by putting a rig in her arm and sharing dirty rigs. And then, he (the client) gets mad at me and says to me, “You’re the one who made it like this.” And I say, “No, it was somebody else.” So he’s infected, and now he wants to give HIV to me. And all I know is that hooking is a waste of time, because he could have HIV. And there are lots of girls at *PEERS* who are HIV+ and they get picked up. And they don’t use condoms, and they get HIV disease, and they could spread it to me—or you—through unprotected sex. Why would I want to get paid for HIV disease? It’s not really worth it. That could affect my looks, my beauty, make me sore.

I think HIV people should be with each other on a different fucking island. They should fuck each other there. Sorry it sounds rude, but some people are so mad when they have diseases and they want to spread them. I know that. (H.B.)

### “Recruitment” to Sex Work and Meth in Youth “Group Housing”

L. J. (*female, 30*), offered a rich narrative of her youth and her first encounters with sex work and meth taking. For L. J. trading sex for meth and money was a part and parcel with being raised in “group home” from the age of 13:

I was addicted to crystal meth, and then I went into a delusional psychosis, because it was bad. And because of that, I lost my eldest child for two months. So, then I just quit drugs after that. Meth was a bad drug. I was so young, in a group home when meth was introduced to me by the older working girls. They were like, “You should try this drug,” and I didn’t even know what it was. I tried it and, of course, I liked it. I did more, and the older girls would teach us, and bring us to johns’ houses, and teach us how to make money. This was the cycle where I was from. All the younger girls were introduced to this cycle by the older girls … It’s really bad for recruitment, like into gang operations and stuff, too. That is what mostly happens in group homes.

STIs were not really discussed. The staff would occasionally get us to do an annual STI checkup. They did encourage the check-up, but we didn’t have to do it. That almost *gave the impression to me that being a sex worker was okay, to a 13-year-old.* We shouldn’t have been having sex. We were just little girls, but we were tested once a year. I thought it was weird that they would do that. It was like the staff didn’t care—or they encouraged it. I know that I wouldn’t want my child at 13 to be having sex. I would do whatever it would take to prevent it.

### Drug Taking by Client, Being Referred to a Potential “Bad Date”

J.P. (*female, 40*) was concerned about HIV risks surrounding drug taking by a prospective *client*—her perceived HIV risks were rooted in the discretion of the “referring” agent for her clients: “Most risky was taking on somebody I really, really didn’t know”.

### HIV Risk from non-commercial, intimate partner’s drug taking

The sharing of oral drug equipment (crack pipes) was identified by H.B. (*female, 30*) as a key concern as an HIV transmission route. One previous male non-commercial, intimate partner was HIV seropositive, and despite engaging in sex with condom use, there were perceived HIV risks:

I had an HIV boyfriend 10 years ago. And he would share pipes with other people, and if you share them, your slobber can give you HIV. I think HIV is in your slobber, and the slobber can’t kill the bacteria. He told me he had HIV, and I said, “I’m not going out with you,” and he said, “Too bad, you’re going out with me, right now”. He put a condom on himself, but I still don’t have diseases—though I still had the risk of getting HIV.

### Client Violence in Relation to Drugs

Many interview participants voiced sexual violence and condomless sex in relation to drug taking. Participant H.B. (*female, 30*), offered an illustrative example. For H.B., many of her clients were met on the streets during a “stroll”. She had previously been working in the sex trade under males who took up the dual role of manager *and* dealer. H.B. was working independently at the time of the interview. H.B. recalled an extremely violent encounter with a client, and she described a lack of investment in her health and safety from her dealers and managers in the aftermath:

When I was young, and I looked like a model, I was prostituted by male dealers because I was young and pretty. One time, this guy grabbed a rock and said, “Let’s go to a field”. And I thought, “This doesn’t sound right”. I went anyway. I should have followed my instincts. It was just forty dollars. Then, I said, “I am not doing this”. He takes me down, spreads my legs, fucks me, picks up a rock and hits me five times over the head, fucks up my model face. I had swollen beats all over my face.

He tried to dig me underground, and he beat me more to bury me more, and he left me there. He raped me, and took my money, and I went back to the drug dealers, and they looked at my face and said, “Who the fuck did this to you?” I said, “Some guy raped me and beat me tonight”. They said, “Oh we’re going to find him right now”. I said “Whatever. You don’t give a shit about girls like me that would get raped and almost killed”. They didn’t even care. They just stood there. There’s no protection for me.

### Distrust of Law Enforcement and Despondence in Reporting Crime

Several participants spoke directly to their previous dissatisfaction with police and law enforcement officials. For instance, R.G. (*male, 30*) adopted an attitude of “why bother reporting”, and he continued: “I mean are the cops going to be there when I need them? When a dude’s stabbing me up in the back alley? … Cops can’t be everywhere at the same time. They just can’t be there”. A.B. (*male, mid-20’s*) shared with criminal self-implication: “We have prostitutes that are criminals now, anyway. I’d probably already be a criminal myself”.

H. B. (*female, 30*) voiced how her interactions with police in Victoria were poor on account of her previous drug taking and drug selling to clients:

There’s are a whole lot of cops in this city, and they don’t like you using drugs outside. And I know that for sure. The dealers all tell me, “Hey, go suck some dick”. Go suck dick and get me money means that I’m not really worth anything. They give me bad, dirty drugs. The cops caught me before, and they stole my drugs. They told me that I had to make a choice, because the cops are either against drugs—or they could be using drugs themselves. Another time I was raped, and they took my drugs.

### HIV Health Literacies and Community Supports

Participants generally voiced how their HIV health literacies had grown more robust over time, and many participants spoke with regret about their previous uptake of practices known to be riskier for HIV infection. The uptake of health promotion discourse was reflected strongly in all participant narratives. For instance, K.D. (*female, 40*) explained:

I think that there are HIV risks, in general, and if you’re knowledgeable, and if you’re not … if you’re care-free … It is equal if you’re a man or a woman, gay or straight … HIV should be addressed, and there should be more information about it. As is said, *when I was young there were no places you could go for information about it, or even a leaflet or booklet*. (my emphasis)

Participants were in consensus agreement that *PEERS* was a valuable community resource. Some participants highlighted the benefits of HIV and ST/BBI testing clinics previously offered on site at *PEERS*. Participants were not asked by the interviewer to comment on *PEERS*, specifically; participants offered favourable feedback of their own volition. For instance, V.R. (*female, 40*) voiced how the community at *PEERS* bolstered her abstinence from drugs:

It was not until I came to *PEERS* that I felt caring … that somebody cared about me. So, I slowly stopped using—and it was mostly cocaine by then—because I was on the Methadone. But, I still used morphine or heroin sometimes, and I am on a low dose of Methadone so that I couldn’t feel it, right. But, then, I slowly stopped using drugs altogether. I felt that somebody cared. Then my kids started to come back around me, and I established my relationship back with them, again.

Participants voiced how condoms were available at no cost at *PEERS*. For instance, as H.B. (*female, 30*) described, “I get condoms here, which is a lot more convenient than going to the store”. L.J. (*female, 30*) was grateful for the counselling and the group activities. K.D., (*female, 40*) a single mother receiving provincial Social Assistance, voiced how the lunch program at *PEERS* were beneficial for reducing monthly expenses for her and her children.

## Discussion

### Drug Taking and HIV Vulnerability

Participants voiced how the extreme needs for drugs and alcohol often drove their engagement in the sex trade wherein violence and (forced) condomless sex ensued. At the structural level, participants’ needs for drugs and alcohol created important barriers to client selectivity and the negotiation of condom use—ultimately exacerbating their risks of HIV infection and transmission (see also Duff et al., 2012; Maher, 1997; Shannon et al., 2008). Jeal, Salisbury, and Turner (2008) have described at length how the street-based sex workers in their study were “trapped” in a cycle of buying and taking drugs.

H.B. (*female, 30*) held rather unique perspectives for HIV infection and transmission. In biomedical and behavioural frames, H.B. was concerned about ingestion of her non-commercial, intimate partner’s “slobber” from his crack pipe as a potential route for HIV transmission. There is evidence that cracked lips (Porter et al., 1993) and chipped glass ends (Porter et al., 1997) on crack pipes increase HIV transmission risks, but the oral cavity remains an extremely uncommon transmission route for HIV (Campo et al., 2006). H.B. also voiced how the riskier injection drug practices of “other” female sex workers increased her risk of HIV infection; H. B. was concerned that “less responsible” sex workers would infect her commercial clients with HIV—who would, in turn, transmit HIV to H. B. This circuitous HIV vector is not implausible, and there is literature on persons’ neoliberal “resistance” of their responsibility for HIV infection in Canada. For instance, Krüsi et al. (2016) have reported how persons living with HIV who inject drugs have insisted on not being “to blame” for their HIV infection due to their previous “safer” injection drug practices. Krüsi et al. (2016) study participants stridently framed their previous risk of HIV infection in terms of environmental risks—rather than in terms of agentic and individualised uptake of HIV preventative practices. Krüsi et al. (2016) ultimately argued that HIV health promotion discourse could focus not only on behavioural intervention, but also on the broader shared and social responsibility for HIV infection and transmission.

### Recruitment to Sex Work

The reasons for initial entry to the sex trade are myriad and complex. While victimisation, poverty (Dodsworth, 2012), the needs for drugs, food, housing, and childcare (Footer et al., 2020), and shifts in the employment sector (Rosen and Venkatesh 2008) could play key roles, for some sex workers, trading sex could have meaning as a career (Murphy and Venkatesh, 2006). There were no indications in the data analysis that participants in this study found sex work a meaningful career choice. Participant L.J. (*female, 30*) voiced intense concerns about growing up vulnerable to ST/BBIs and HIV in a “group home” environment wherein the older youth recruited L.J. for sex work and meth taking. This is not an uncommon macro-narrative: For instance, in a study of 255 street-based female sex workers in Vancouver, Shannon et al. (2011) found meth an important factor within intimate drug using sex partnerships, “suggesting a gendered pattern of risk colliding along the intersections of street-based sex and drug markets” (p. 79). In Footer et al. (2020) study, 21% of female sex workers below the age of 18 reported being coerced, threatened, pressured, misled, tricked, or physically forced into sex trade entry; this compares with only 4% of women reporting similar coercion who entered the sex trade above the age of 18. Benoit et al. (2017b) found three interwoven structural and agentic components to sex trade entry in Canada: critical life events, the need and desire for money, and the personal appeal of sex work. There are indications in L.J’s narrative that her entrance to a “group home” in her youth would constitute a critical life event.

### Sex Workers in Relation to Police and Policing

Police and sex workers have a complex relationship. In alignment with findings by Krüsi et al. (2014), some participants in the present study expressed a deep mistrust of police. In H. B.’s (*female, 30*) narrative, previous caustic relations with the police in Victoria were tightly woven with drug taking and drug selling. The repressive policing of sex workers and their clients deprioritises the health, safety, and rights of sex workers, antagonising legal due process (Platt et al., 2018). Following one assault and “rape”, the police were not perceived by participant H. B. as a viable (and reliable) recourse for justice. Sex workers in Canada fear telephoning for police assistance in cases of emergency because sex workers fear detention (themselves, their colleagues, and their management) (Crago et al., 2021). H. B., alongside participant A. B., voiced how they were on the receiving end of powerful and ongoing stigma and discrimination from police (see Benoit et al., 2016). When reflecting on contacting the police, A. B. voiced how he would potentially “already be a criminal, anyway”, reflecting his self-identified dual role as both “victim” and “victimiser” to police, as previously described by Krüsi et al. (2016).

There are suggestions that sex workers need unconstrained access to police and emergency services, police protection, and the ability to report clients and client environments that threaten their safety (Crago et al., 2021). Participant R.G. (*male, 30*) voiced how he did not feel the police were an immediate and helpful recourse, adopting the attitude akin to “Why *bother* involving the police?” The findings here highlight above all the important barrier and caustic relationship created between sex workers and police by drug involvement, particularly drug involvement interwoven with street-based (outdoor) sex work in Victoria.

### The Support of ‘PEERS’ and HIV Health Literacies

There is a robust literature on the beneficial role of community health supports and networks for sex workers (Blanchard et al., 2013; Benoit et al., 2017a; Goldenberg et al., 2020). Participants in the present study generally voiced how the *PEERS* community in Victoria provided a “safer space” for accessing social supports and safer sex supplies. Participants also shared with how *PEERS* had previously hosted beneficial on-site HIV and ST/BBI testing. There are indications in the interview data that valuable HIV health promotion discourse concerning condom use and safer drug injection practices had been reaching participants. In the literature, HIV health literacies have been previously identified as “lower” among sex workers (Tokar et al., 2018) alongside other vulnerable populations across a variety of settings (for instance, Johnston et al., 2011; Budhwani et al., 2017), and these HIV literacies have also been found lower in rural versus urban areas (Veinot and Harris 2011). I modestly report HIV literacies concerning HIV *behavioural prevention* in the sample as more than “adequate”; HIV literacies concerning HIV treatment prognoses and PrEP could however have been more robust.

Participants voiced intense fear, anxiety, and stigma surrounding HIV infection throughout the interviews. Participants often used the terms *AIDS* and *HIV* interchangeably to denote a “death sentence”. These general attitudes align with the “AIDS fatalism” and “HIV treatment uncertainty” paradigms previously described by Conroy et al. (2013). These attitudes bolster support for intensifying dissemination of the more novel “Undetectable equals Untransmittable” (or U = U) HIV messaging (York, 2019; Eisinger et al., 2019) for sex worker community health outreach and programming. HIV treatment prognosis is presently more promising than identified and described by study participants: For instance, in the province of BC at age 20, the health-adjusted life expectancy is roughly 31 years for men, and 27 years for women (Hogg et al., 2017). The fatalistic narratives were however strong. By contrast, R. G. (*male, 30*) explained:

I am not just going to watch this old man get his way, and fuck him, and then I end up having to live with the knowledge that I am going to die … why would I put myself at risk of a deadly disease without a cure?

One possible origin of this HIV “death sentence” discourse could be from participants’ previous and ongoing social immersion with persons living with HIV on the more vulnerable end of the social and economic spectra, persons experiencing difficulties with antiretroviral adherence (for instance, because of street entrenchment) such that participants would infer and conclude that HIV treatment prognoses remained poorer. For added perspective, one recent HIV cohort study found that the leading cause of death of persons living with HIV in the province of BC was drug overdose (Salters et al., 2021).

### On PrEP

Male participants in this study could benefit from no cost PrEP as a structural HIV preventative intervention in BC. PrEP is presently available at no cost for eligible persons province wide from the *British Columbia Centre for Excellence in HIV/AIDS*. Cis-female participants who share injection drug equipment with persons living with HIV would also be eligible for no cost PrEP. I will not duplicate here the literature and discussions on PrEP treatment and acceptability (Knight et al., 2016; Tan et al., 2017; Mosley et al., 2018; Lee-Foon et al., 2020). There is however limited but real precedent for indicating PrEP for cis-female sex workers in Canada: For instance, O’Byrne et al. (2019) have recommended PrEP through a “nurse-led active-offer” PrEP service in Ontario for one exemplar female sex worker at high exposure risk for HIV (but mid-range for sexual practices) in their study—from a “case-by-case” approach for administering PrEP.

At the periphery of the scope to this study, the novel uptake of PrEP by sex workers would not be without caveat. The differential uptake of PrEP in the sex worker community could catalyse the de-stratification of sex workers’ incomes in relation to condomless sex, that is, potentially driving down the cost of condomless sex for all sex workers. Increased PrEP uptake among *some* sex workers could also normalise condomless sex in the sex trade for all sex worker, irrespective of their uptake of PrEP. This could be of consideration for health implementation scholars should PrEP become available at no cost to survival sex workers.

## Conclusion

### Study and Limits

Participants revealed important contexts and conditions under which they were vulnerable to HIV infection, both occupationally as well as in their personal lives and lived experiences. At the agentic and interpersonal levels, participants were keenly aware of how HIV could be transmitted (condomless sex and sharing drug equipment), yet participants voiced strongly how structural and systemic features (for instance, client violence, the need for drugs, and “bad date” referrals) could squeeze and constrain their agency to take up safer practices, mediating their optimal HIV health and safety. HIV/AIDS fatalistic attitudes among participants could potentially be addressed presently by dovetailing with the greater “Undetectable equals Untransmittable” (U = U) project aimed at de-stigmatising living with HIV and promoting the sexual citizenship of persons living with HIV—inclusive of presently promising HIV treatment outcomes. In the face of intense HIV vulnerability voiced by participants in the present study, I argue that an expansion of eligibility for no cost PrEP for cis-female sex workers in the province of BC could be among the more important structural shifts to curb the transmission of HIV to survival sex workers.

The study has limits. There could be a constraint to the study’s validity on account of the duration of time from data collection to publication date. The conclusions of this study were reached from analysis of interview data generated by a smaller (*n* = 9) sample of study participants. This sample size in ths study reflects on one hand fidelity to the grounded theory method’s theoretical saturation; on the other hand, the smaller sample reflects constraints inherent with the recruitment and practical availability of persons comprising the difficult-to-reach population of survival sex workers in Victoria, Canada. The eventual sample size of this qualitative study however supported a greater depth of grounded analysis in comparison with a quantitative study of similar nature. In terms of sampling demographics, women and persons with Indigenous ancestry were over-represented; the sample was not however inconsistent with the demographic make-up of survival sex workers in Canada (Benoit et al., 2014). There was a social desirability bias: Participants could have over-emphasised their condom use, and under-emphasised their sharing of drug equipment. Member-checking was impossible on account of the anonymous study participation. The sample was biased towards vulnerable sex workers who were clients at a non-profit outreach organisation. Sex workers unwilling to “out” themselves in accessing specialised sex work services, persons new to sex work, and persons otherwise disenchanted by sex worker outreach would have been excluded in the convenience sampling procedure.

### Future Research on the (de-)criminalisation of Sex Work

The present study was delimited to a sample of survival sex workers living without HIV or uncertain of their HIV status. Canada’s *Bill C-36* was signed into law in November of 2014, targeting the “purchasers” of sex, and to date, there remain no indications in the literature how this law may be mediating the reporting of potential crimes *undertaken by sex workers* surrounding sex transactions; I speculate outside the scope of the present study that clients would presently fear that they, themselves, could face criminal arrest in the reporting of “crimes” undertaken by sex workers. If and when sex work is fully de-criminalised in Canada, I anticipate an influx in the criminal reporting “of” sex workers including, but not limited to, HIV nondisclosure. In a strategic reversal, future research could investigate whether there are potential *protective* legal benefits afforded to survival sex workers from the criminalisation of the “demand” side within the Canadian sex trade.

## Data Availability

The datasets presented in this article are not readily available because a small, close-knit community of sex workers from this study’s community setting could be identified by the greater dataset. Requests to access the datasets should be directed to brytt@uvic.ca.

## Appendix 1

Thematic Interview Guide

Intimacy and Risk among Sex Workers in the Context of HIV Infection

Themes to be explored:

1) In work life and in personal life, explore participants’ thoughts about the risk of becoming HIV+
2) Explore how participants minimize the risk of becoming HIV+
3) Explore how, or whether, the risks of HIV affect intimate relationships, with boyfriends, girlfriends, clients, and others
4) Explore participants’ thoughts on people being obligated to tell their sexual partners that they are HIV+, and how these thoughts might be influenced by condom use or taking antiviral medications to suppress the virus
5) Explore participants’ knowledge of laws which make HIV disclosure a legal obligation
6) Explore how, or whether, participants would feel safer knowing HIV disclosure laws exist
